# COVID-19 and Plasmodium vivax malaria co-infection

**DOI:** 10.1016/j.idcr.2020.e00879

**Published:** 2020-06-20

**Authors:** Sundus Sardar, Rohit Sharma, Tariq Yousef Mohammad Alyamani, Mohamed Aboukamar

**Affiliations:** a Department of Internal Medicine, Hamad Medical Corporation, Doha, Qatar; b Department of Infectious Disease, Hamad Medical Corporation, Doha, Qatar

**Keywords:** SARS-CoV-2, COVID-19, Malaria, *Plasmodium vivax*, Co-Infection, Artesunate

## Abstract

The ongoing outbreak of COVID-19 poses an unprecedented global health challenge. With its variety of clinical manifestations including, but not limited to, fever, cough, diarrhea, vomiting, headache, myalgia and fatigue, it may be challenging to distinguish COVID-19 from a spectrum of diseases with similar presentations, such as malaria, especially in endemic areas. Risk of concomitant infections also remains a concern owing to overburdening of healthcare services and possible scarcity of resources. We present the first reported case of confirmed COVID and malaria co-infection. In this case, we emphasize the need for vigilance from frontline clinicians for timely diagnosis and appropriate clinical management of potential co-infections in the COVID era.

## Highlights

• Co-infection with COVID-19 and mosquito-borne diseases such as malaria and dengue is of clinical importance in endemic regions.
• Symptoms of COVID-19 overlap with many infectious diseases caused by a variety of pathogens.
• Early identification of co-infections with COVID-19 is essential for appropriate and timely management, especially in regions where syndemics may occur.

## Introduction

The coronavirus infection 2019 (COVID-19), which is caused by SARS-CoV-2, emerged in Wuhan, China in December 2019 and has since reached pandemic proportions affecting more than 8 million cases worldwide with total deaths exceeding 400,000 [1]. Typical presentations include a spectrum of clinical manifestations, mainly with respiratory involvement, with some cases exhibiting cardiac, gastrointestinal, neurological or renal manifestations. Recent reports have emerged on co-infections in COVID-19 with bacterial, viral, and fungal pathogens. We present the case of a previously healthy young man, diagnosed with COVID-19 and malaria infection, treated with artesunate. To the best of our knowledge, this is the first reported case of malaria and COVID-19 co-infection. It emphasizes the necessity to focus on other causes of secondary infection, which may significantly alter the course of clinical management and prognosis in concurrence with SARS-CoV-2 infection. Despite unprecedented times, clinicians must approach the cases with a holistic approach focusing on history, physical exam and investigations while keeping in mind a broad differential diagnosis for fever wherever clinically relevant.

## Case report

A 34-year-old man, with no previously diagnosed chronic medical conditions, presented to the hospital with a three-day history of fever, myalgia and vomiting, associated with right upper quadrant abdominal pain. He denied any cough, shortness of breath, or other respiratory symptoms. He had no history of diarrhea, constipation or genitourinary symptoms. He had a travel history to Pakistan three months prior to the current presentation and was febrile with a temperature of 39.6 °C, blood pressure of 109/72 mmHg, tachycardic with a heart rate of 148 beats per minute, tachypneic with a respiratory rate of 24 breaths per minute, with oxygen saturation of 97 % on room air.

Physical examination revealed a jaundiced man with tenderness in right hypochondrium with no guarding. Laboratory investigations were significant for leukopenia with white blood cell count 3.6 × 10^3^/μL (reference range: 4.0–10.0 × 10^3^/μL), lymphopenia 0.2 × 10^3^/μL (reference range: 1.0–3.0 × 10^3^/μL), hemoglobin 14.5 g/dL (13.0–17.0 g/dL) and thrombocytopenia with platelet count 30 × 10^3^/mL (reference: 150–400/mL), elevated LDH 481 U/L (reference: 135−225U/L) and low haptoglobin < 10 mg/dL (30−200 mg/dL). Further investigations revealed direct hyperbilirubemia [total bilirubin 7.66 mg/dL (reference range: 0.30–1.23 mg/dL) with direct bilirubin 4.27 mg/dL (reference: 0−0.29 mg/dL)], C-reactive protein 238 mg/L (reference: 0−5 mg/L), procalcitonin 61.30 ng/L (reference: 0.5–2.0 ng/L), lactic acid 2.4 mmol/L (reference: 0.5–2.2 mmol/L), elevated ferritin level 1,831.0 μg/L (reference: 48–420 μg/L) and high d-dimer 5.40 mg/L (reference: 0.00−0.44 mg/L). The chest X-ray was unremarkable. The electrocardiogram showed sinus tachycardia.

Ultrasound of the abdomen revealed hypoechoic liver with prominent periportal tracts and no evidence of cholelithiasis. Malaria smear was positive for *Plasmodium vivax* with a parasitemia of 1.2 % with concomitant positive nasopharyngeal PCR for SARS-CoV-2. Initial treatment with artemether-lumefantrine was initiated with fluid resuscitation. Due to vomiting, artemether-lumefantrine was discontinued and intravenous artesunate was administered at a dose of 2.4 mg/kg at 0, 12, 24, and 48 hours. Once the patient was able to tolerate oral therapy, anti-malarial treatment was continued with artemether-lumefantrine. Repeated ultrasound of the abdomen revealed an interval decrease in the periportal echogenicity and normal echotexture of the hepatic parenchyma.

Upon discharge, laboratory investigations revealed normal white blood cell count 6.8 × 10^3^/μL, hemoglobin 13.6 g/dL, improving platelet count 144 × 10^3^/μL, and total bilirubin 1.35 mg/dL. During the hospital course, the patient did not develop any respiratory symptoms nor required any oxygen supplementation. The patient was discharged to a quarantine facility to complete a 14-day isolation and a primaquine prescription for treatment of *P. vivax* relapse. Repeated malaria smear after 5 days from initial presentation revealed clearance of parasitemia. COVID-19 nasopharyngeal PCR repeated 7 days after admission was confirmed as negative.

## Discussion

Currently, healthcare systems worldwide are enduring unprecedented strains and physicians continue to play a pivotal role in prompt identification and pertinent clinical management in the COVID-19 pandemic. It is important to note that many symptoms of COVID-19 such as fever, myalgia, and headache are similar to the co-infections which may occur concurrently with SARS-CoV-2 infection. Measures must be taken in patients with overlapping symptoms to identify such cases. To our best of our knowledge, this is the first reported case of malaria with SARS-CoV-2 infection. Coinfection with malaria in endemic regions might be of high clinical importance as the pandemic continues to spread.

Co-infections in COVID-19 are relatively unexplored with recently emerging data reporting concomitant infections with influenza [[2], [3], [4], [5], [6], [7]], varicella-zoster [8,9], and respiratory syncytial virus (RSV) [10]. However, less prevalent than in patients with influenza infection, some cases of bacterial co-infection with *Mycoplasma pneumoniae, Pseudomonas aeruginosa, Hemophilus influenzae*, and *Klebsiella pneumoniae* have also been reported in patients with COVID-19 [10]. A recent study concluded that the prevalence of secondary infections with COVID-19 amongst non-survivors might be as high as 50 % [11]. Owing to similar clinical presentations, recent concerns regarding dual outbreaks of COVID-19 and dengue have surfaced [12], thereby, physicians must be vigilant to identify and appropriately manage cases in such syndemics in a timely manner.

In our case, the source from which malaria infection was contracted remains suspicious, with a plausible explanation suggesting re-activation of *Plasmodium vivax* from previous infection with dormant liver stage parasites or hypnozoites. Other possible mechanisms may include local transmission of malaria parasite via Anopheles mosquito which cannot be ruled out completely. Qatar is not a malaria-endemic region but it does cater to a significant percentage of the population that travels or immigrates from endemic parts of the world. It is worthwhile to note that in many cases severe manifestations of malaria may be due to heightened proinflammatory response, the same may be true in many cases of COVID-19. Malaria and COVID-19 co-infection could then lead to excessive pro-inflammatory response leading to severe manifestations and poor prognosis [13].

The treatment regimen for COVID-19 pandemic yet remains undefined, with a myriad of clinical trials exploring the role of multiple medications as a possible cure. While recent studies have refuted the benefit of hydroxychloroquine or chloroquine for the treatment of COVID-19, concerns have arisen regarding possible consequences of mass consumption of these medications in endemic malaria settings [14]. Artemisinins such as artesunate have lately attracted much attention in the management of COVID-19, owing to their anti-viral and anti-inflammatory properties, likely attributed to inhibition of Nuclear Factor kappa B (NF-kB) downregulation and consequent disruption of viral replication in the early phase [15].

In this case, artesunate and artemether were initiated as the treatment regimen; whether these agents offered protective effects from respiratory deterioration or multi-organ involvement despite SARS-CoV-2 infection is unclear and should be further explored. Additional studies are also required to investigate whether patients with concurrent infections have a worse prognosis as compared to those with SARS-CoV-2 as the sole detected pathogen.

## Conclusion

Our case highlights the importance of identifying possible underlying secondary infections in concurrence with SARS-CoV-2, which may be otherwise overlooked amidst the challenges of the current unprecedented COVID-19 pandemic. It is essential for physicians to remember that neither co-infection with pathogens can be ruled out when COVID-19 is confirmed nor positive test for other pathogens completely negates the presence of COVID-19 co-infection. Our case of COVID-19 and malaria co-infection was treated with artemisinins and recovered successfully. Additional trials are required to investigate the role of artemisinins in the management of SARS-CoV-2 infection. Further studies are warranted to explore the risk factors, clinical outcomes, challenges in management, and prognosis of cases with COVID-19 and co-infection with another pathogen.

## Consent

Written informed consent was obtained from the patient for publication of this case report. A copy of the written consent is available for review by the Editor-in-Chief of this journal.

## Funding

The publication of this article was funded by the Qatar National Library.

## Author contributions

Writing the initial draft of the manuscript: Sundus Sardar, Rohit Sharma

Conceptualization and supervision: Mohamed Aboukamar

Medical management of the case: Tariq Yousef Mohammad Alyamani, Mohamed Aboukamar

Revising the manuscript critically and literature review: Sundus Sardar, Rohit Sharma, Tariq Yousef Mohammad Alyamani, Mohamed Aboukamar

The first authors (SS and RS) contributed equally to the writing and preparation of this article. SS and RS have written the initial draft of the manuscript and performed the literature review. The draft was revised and updated by SS and RS with supervision from MA. TYMA and MA were part of the medical treating team. All the authors critically reviewed the initial and the final draft of the manuscript and approved it for submission.

## Declaration of Competing Interest

The authors have no conflict of interest relevant to this case.
